# Integrated analysis of fecal microbiota and metabolomics reveals unique characteristics of asymptomatic and classic celiac disease

**DOI:** 10.3389/fmicb.2025.1636007

**Published:** 2025-11-13

**Authors:** Shanxia Yao, Shenglong Xue, Na Li, Tian Shi, Yan Feng, Munila Maimaiti, Ayinuer Maimaitireyimu, Halina Halike, Feng Gao

**Affiliations:** 1 College of Life Science and Technology, Xinjiang University, Urumqi, China; 2 Department of Gastroenterology, People’s Hospital of Xinjiang Uygur Autonomous Region, Urumqi, China; 3 Xinjiang Clinical Research Center for Digestive Diseases, Urumqi, China

**Keywords:** asymptomatic celiac disease, classic celiac disease, microbiota, metabolites, diagnostic markers

## Abstract

**Introduction:**

Celiac Disease (CeD) is an autoimmune small intestinal disorder triggered by gluten, with clinical subtypes including typical, atypical, and asymptomatic forms.While classic CeD development is linked to microbes/metabolites, their relationships with other subtypes remain unclear.

**Methods:**

We conducted 16S rDNA sequencing on fecal samples from 14 asymptomatic CeD (SCeD) patients and integrated this data with our team’s prior sequencing data of 30 classic CeD (CDF) patients and 30 healthy controls (CDFH).

**Results:**

16S rDNA results showed: Compared to CDFH, SCeD had lower abundances of Bacteroides, Alistipes, CAG-352 and higher abundances of Blautia, Collinsella, Dorea, Mediterraneibacter, Gemmiger; a random forest model based on 8 differential microbes distinguished SCeD from CDFH (AUC = 0.97). Compared to CDF, SCeD had lower Bacteroides and higher Faecalibacterium, Blautia, Collinsella, Agathobacter—suggesting Bacteroides may relate to CeD symptoms, while Faecalibacterium and Agathobacter may alleviate symptoms. Metabolomic analysis identified differential metabolites between SCeD and CDFH (enriched in “Steroid Hormone Biosynthesis,” “Primary Bile Acid Biosynthesis,” “Tryptophan Metabolism” via KEGG) and between SCeD and CDF (enriched in “Tryptophan Metabolism,” “Biosynthesis of Plant Secondary Metabolites,” “Degradation of Flavonoids”). Spearman analysis showed correlations between differential microbes and metabolites.

**Discussion:**

In conclusion, different CeD subtypes may involve a “host-microbe-metabolite” trinity network: A random forest model built with SCeD-CDFH differential microbes/metabolites is a high-efficacy SCeD diagnostic tool; modulating these microbes/metabolites could be a new entry point for CeD mechanism research and adjunctive therapy.

## 1 Introduction

Celiac Disease (CeD) is an autoimmune enteropathy triggered by exposure to dietary gluten in genetically susceptible individuals (Catassi et al., 2022). The global prevalence of CeD is approximately 1.4%, and recent studies indicate a rising trend in its prevalence—posing a significant impact on patients’ quality of life and overall health (Lebwohl and Rubio-Tapia, 2021). CeD presents with a wide range of clinical manifestations, most commonly gastrointestinal symptoms such as abdominal pain and diarrhea; additionally, patients with CeD face an increased risk of developing complications affecting other bodily systems (Catassi et al., 2022). However, a subset of patients show no obvious clinical symptoms and are therefore classified as having asymptomatic celiac disease (SCeD). As a distinct subtype of CeD, SCeD is defined by the absence of overt clinical symptoms, alongside positive serological test results and pathological changes in the small intestinal mucosa (Marsh grade ≥ 2). Notably, SCeD patients remain at risk of progressing to classic celiac disease (CDF) or developing other related complications (Laurikka et al., 2022). Given the heterogeneity of CeD subtypes, analyzing only a single subgroup makes it challenging to address complex research questions. Timely intervention after diagnosis, for example, strict adherence to a gluten-free diet (Gluten-Free Diet, GFD), can effectively curb the progression of the disease and prevent the occurrence of complications (Aljada et al., 2021). That said, due to the lack of typical symptoms in SCeD, conventional diagnostic approaches often lead to missed diagnoses. Early detection of SCeD is critical for preventing complications like malnutrition, osteoporosis, and elevated risk of malignant tumors; delayed diagnosis resulting from missed cases significantly increases the likelihood of these adverse outcomes. Thus, novel diagnostic strategies are urgently needed to enhance the diagnostic efficiency of SCeD (Makharia et al., 2022). Ultimately, understanding the differences in microbial communities and metabolite profiles across various CeD subtypes can facilitate both the diagnosis and adjuvant treatment of CeD.

The intestine, a digestive organ consisting of the large and small intestines, is often referred to as “the human body’s second brain.” As a key “microbial organ” in humans, the gut microbiota plays a pivotal role in maintaining intestinal homeostasis, regulating immune responses, and facilitating nutrient metabolism (Zhao et al., 2023). Studies have shown that gut microbial dysbiosis is associated with various gastrointestinal disorders (Li H. et al., 2023); it produces diverse metabolites—such as bile acids, short-chain fatty acids, tryptophan, and methane—all of which are critical for intestinal peristalsis and secretion (Fan et al., 2022). Moreover, research has demonstrated that gut microbial dysbiosis is closely associated with the development and progression of multiple autoimmune diseases, including CeD (Han et al., 2025). Furthermore, CeD development is shaped by genetic background, diet, and environment—and these same factors also modulate the gut microbiota (Gupta et al., 2023). Specifically, research focusing on patients with classic celiac disease (CDF) has confirmed that, when compared to healthy controls, CDF patients show marked changes in gut microbial community structure. Such changes include higher abundances of specific pathogenic bacteria and lower abundances of beneficial bacteria. These microbial shifts are not only directly linked to intestinal mucosal barrier damage but also influence immune-inflammatory responses through metabolite regulation, ultimately driving the pathological initiation, progression, and symptom onset of CDF (Bascuñán et al., 2025; Catassi et al., 2024). Certain bacteria elicit host immune responses by expressing epitopes analogous to gliadin (Belei et al., 2023), whereas other microbes induce intestinal mucosal damage by disrupting host immune responses (Acharya et al., 2024), ultimately contributing to CeD’s clinical manifestations. In contrast, specific beneficial microbes can alleviate disease symptoms by preserving intestinal microecological balance (Peng et al., 2022). Alterations in gut microbiota composition typically result in shifts in metabolite profiles (Koh and Bäckhed, 2020). Metabolomics allows for comprehensive analysis of endogenous metabolite alterations in biological systems, providing insights into an organism’s physiological and pathological status (Rostami-Nejad et al., 2024). In CeD research, metabolomic technologies have been used to identify potential biomarkers and uncover disease-related metabolic pathways (Girdhar et al., 2023). Early studies conducted metabolomic analyses on samples from CD patients and healthy controls (CDH), revealing a series of disease-associated differential metabolites involved in multiple metabolic pathways such as energy metabolism, amino acid metabolism, and lipid metabolism. These findings provided new insights into the pathogenesis of celiac disease (Kelley et al., 2025). Additionally, metabolomics has been applied to analyze changes in metabolites in CeD patients before and after a gluten-free diet (GFD) (Vacca et al., 2022). However, these studies have primarily focused on celiac disease with typical symptoms, while the characteristics of the microbial community, metabolic alterations, and their interactions in SCeD patients remain unclear. Currently, there is still a lack of reported research on the microbiota and metabolites in SCeD patients.

Combining the use of 16S rDNA sequencing and untargeted metabolomics enables an understanding of disease pathogenesis from two perspectives: the gut microbial community and metabolite levels (Zhao et al., 2025). 16S rDNA sequencing can accurately characterize changes in the composition and structure of the gut microbial community, while untargeted metabolomics can capture disease-related metabolite fingerprint profiles. The combination of these two techniques helps reveal the interaction between microbes and host metabolism, providing more comprehensive insights into the pathogenesis of celiac disease (CeD) (Gu et al., 2024). Therefore, based on 16S rDNA sequencing and untargeted metabolomics, this study aims to investigate the roles of microbes and metabolites in different subtypes of CeD. Specifically, by comparing differences in microbes and metabolites between the asymptomatic CeD (SCeD) group and the healthy control (CDFH) group, we seek to identify microbes and metabolites with diagnostic value for SCeD. A diagnostic model will then be developed using machine learning, providing new methods and tools for the early diagnosis of SCeD. Additionally, by analyzing differences in microbes and metabolites between the SCeD group and the classic CeD (CDF) group, this study explores the roles of microbes and metabolites in the development of different CeD subtypes. This work not only provides a theoretical basis for a deeper understanding of the progression of different CeD subtypes but also lays the groundwork for the development of new therapeutic strategies.

## 2 Materials

### 2.1 Patients and healthy controls

Starting in 2022, the Gastroenterology Department of the People’s Hospital of Xinjiang Uygur Autonomous Region initiated an epidemiological survey. A total of 5,600 individuals participated in this investigation. Through screening, 54 patients tested positive for tTG were identified. Based on the questionnaires, 14 individuals who exhibited no clinical symptoms but had tTG levels greater than 200 were included in the SCeD group. Additionally, 30 patients with typical CeD diagnosed in our team’s earlier study were included as the CDF group, along with 30 healthy controls (CDFH group) who were negative for both EMA and tTG antibodies, matched by ethnicity, gender, and age (±3 years) (Shi et al., 2022). Thus, this study included a total of 44 cases in the disease group (CeD) (14 SCeD cases with complete epidemiological survey information and stool samples, and 30 CDF cases) and 30 cases in the healthy control group (CDFH), All participants included in this study are from the Xinjiang Uygur Autonomous Region of China. The inclusion and exclusion criteria for this study are as follows:

Inclusion criteria: CDF group: Positive for serum tissue transglutaminase IgA (tTG-IgA) or endomysial antibody IgA (EMA-IgA), with celiac disease (CeD) confirmed by small intestinal biopsy combined with histopathological diagnosis (Bai and Ciacci, 2017). SCeD group: Serum tTG-IgA titer 10 times the normal value, positive for EMA-IgA, and exempt from biopsy for direct diagnosis of CeD (Rubio-Tapia et al., 2023). Healthy control group: Negative for serum tTG-IgA or EMA-IgA, on a normal diet, and no chronic diseases in recent periods.

Exclusion criteria: Patients with other autoimmune diseases (including inflammatory bowel disease [IBD], autoimmune gastritis, type 1 diabetes mellitus [T1DM], and rheumatoid arthritis [RA]); Patients with other specific gastrointestinal infections (bacterial or viral infections) or parasitic infections; Pregnant or lactating women; Patients who have received antibiotic or probiotic treatment recently; Patients who are unwilling to participate in this study. Patients meeting any of the above criteria will be excluded from the study.

All participants in this study signed the informed consent form. This study was approved by the Ethics Committee of the People’s Hospital of Xinjiang Uygur Autonomous Region, with the Ethics Approval Number: KY20220311067.

## 3 Methods

### 3.1 16S rDNA sequencing and data processing

Total genomic DNA was extracted from stool samples of the enrolled population using the FastPure Stool DNA Isolation Kit (MJYH, Shanghai, China) according to the manufacturer’s instructions. DNA integrity was assessed by 1% agarose gel electrophoresis, and concentration and purity were determined using the NanoDrop2000 (Thermo Scientific, United States). Using the extracted DNA as a template, PCR amplification of the 16S rRNA gene V3-V4 variable region was performed with the barcoded upstream primer 338F (5’-ACTCCTACGGGAGGCAGCAG-3′) and downstream primer 806R (5’-GGACTACHVGGGTWTCTAAT-3′) (Marascio et al., 2023). Libraries were constructed using the NEXTFLEX Rapid DNA-Seq Kit, followed by adapter ligation, magnetic bead selection, PCR amplification, and magnetic bead recovery. Sequencing was performed on the Illumina NextSeq 2000 platform. Raw sequencing reads underwent quality control and assembly using fastp (Chen et al., 2018) (version 0.19.6) and FLASH (Magoč and Salzberg, 2011) (version 1.2.11). Reads were filtered for low-quality bases, short reads, and N-containing bases as required. Sequences were assembled based on overlap relationships and screened, with orientation adjusted according to barcodes and primers. OTUs were clustered at 97% similarity using UPARSE v7.1 (Edgar, 2013), and chimeras were removed. Sample sequences were downsampled to 20,000 sequences, achieving an average sequence coverage of 99.09% post-downsampling. OTU species taxonomic annotation was performed using RDP classifier (version 2.11) with a confidence threshold of 70%. Functional prediction analysis of 16S rRNA genes was conducted using PICRUSt2 (Douglas et al., 2020) (version 2.2.0).

### 3.2 Sample preparation, sequencing, and data processing for LC–MS

#### 3.2.1 Solid sample processing

Place 50 mg of solid sample into a 2 mL centrifuge tube. Add one 6 mm diameter grinding bead. Extract metabolites using 400 μL of extraction solution (methanol:water = 4:1) for metabolite extraction. Grind for 6 min at −10 °C and 50 Hz using a cryogenic tissue grinder. Perform low-temperature ultrasonic extraction at 5 °C and 40 kHz for 30 min. After standing at −20 °C for 30 min, centrifuge at 4 °C and 13,000 g for 15 min. Transfer the supernatant to vials with inserts for instrument analysis. Equal volumes of metabolites from all samples were pooled to create QC samples. During instrument analysis, one QC sample was inserted every 5–15 samples to assess analytical process repeatability.

The analysis was performed on a Thermo Fisher Scientific Ultra High Performance Liquid Chromatography-Quadrupole-Tandem Mass Spectrometry system (UHPLC-Q Exactive HF-X) (Shanghai Meiji Biotechnology Co., Ltd.). Chromatographic conditions were as follows: 3 μL of sample was injected and separated on an HSS T3 column (100 mm × 2.1 mm i.d., 1.8 μm). Mobile phase A: 95% water + 5% acetonitrile (containing 0.1% formic acid); Mobile phase B: 47.5% acetonitrile + 47.5% isopropanol + 5% water (containing 0.1% formic acid). Flow rate: 0.40 mL/min; Column temperature: 40 °C. Mass spectrometry employed both positive and negative ion scanning modes. The mass scan range was 70–1,050 m/z. Sheath gas flow rate was 50 psi, auxiliary gas flow rate was 13 psi, auxiliary gas heating temperature was 425 °C, positive mode ion spray voltage was 3,500 V, negative mode −3,500 V, ion transfer tube temperature 325 °C, normalized collision energy 20–40–60 V cycle, MS^1^ resolution 60,000, MS^2^ resolution 7,500, data acquired in DDA mode.

Raw data were imported into Progenesis QI software for baseline filtering, peak identification, and other processing steps, yielding a data matrix containing retention times, mass-to-charge ratios, and peak intensities. Metabolite information was obtained by matching MS and MS/MS data against the HMDB, Metlin, and Meiji’s proprietary databases. The data matrix was uploaded to the Meiji Cloud platform, where it underwent the following preprocessing steps: 80% rule for missing value imputation, sum normalization, removal of QC samples with RSD > 30%, and log10 transformation. PCA and OPLS-DA analyses were performed using the R package ropls. Model stability was assessed through 7-fold cross-validation. Significantly different metabolites were identified based on VIP > 1 in the OPLS-DA model and *p* < 0.05 in Student’s t-tests. Finally, pathway annotation of differential metabolites was performed using the KEGG database, followed by pathway enrichment analysis with the Python scipy.stats package. Fisher’s exact test was applied to identify relevant biological pathways.

### 3.3 Statistical analysis

All data analyses were conducted on the Majorbio Cloud Platform.^1^ Alpha diversity analysis: The mothur software (Schloss et al., 2009) was used to calculate Alpha diversity indices (e.g., Chao 1, Shannon), and the Wilcoxon rank-sum test was applied to analyze differences in Alpha diversity across groups. Microbial community structure analysis: Principal Coordinate Analysis (PCoA) based on the Bray-Curtis distance algorithm, combined with the PERMANOVA non-parametric test, was used to analyze the similarity and differences in microbial community structure among samples. Screening of differentially abundant bacterial taxa: Linear Discriminant Analysis Effect Size (LEfSe) analysis (Barberán et al., 2012) (with thresholds: LDA > 2, *p* < 0.05) was performed to identify bacterial taxa with significant differences in abundance between groups. Impact of clinical indicators: Distance-based Redundancy Analysis (db-RDA) was used to investigate the influence of clinical indicators on gut bacterial community structure; linear regression analysis was employed to evaluate the effect of key clinical indicators on microbial Alpha diversity indices. Correlation network analysis: Species were selected for correlation network analysis [80] based on Spearman correlation analysis (with thresholds: |*r*| > 0.6, *p* < 0.05). Differences among multiple groups were analyzed using the Kruskal-Wallis test, while comparisons between two groups (CeD-CDFH, SCeD-CDFH, SCeD-CDF) employed Student’s *t*-test. Differences were considered statistically significant at *p* < 0.05. Differential metabolite analysis within each subgroup was screened using *p*-values and Variable Importance Projection (VIP) scores, defining metabolites with *p* < 0.05 and VIP > 1 as differentially altered. All significantly altered metabolites or bacterial species were incorporated into a random forest model, with highly important microbes and metabolites used to construct diagnostic models. Receiver operating characteristic (ROC) curves were plotted using R software packages, and area under the curve (AUC) was calculated to evaluate predictive model performance. Pearson correlation analysis was employed to assess correlations between differential metabolites and differential microbial communities, with analyses conducted at the genus level.

## 4 Results

### 4.1 CeD-induced gut microbiota alterations based on 16S rDNA data

**Figure 1:**
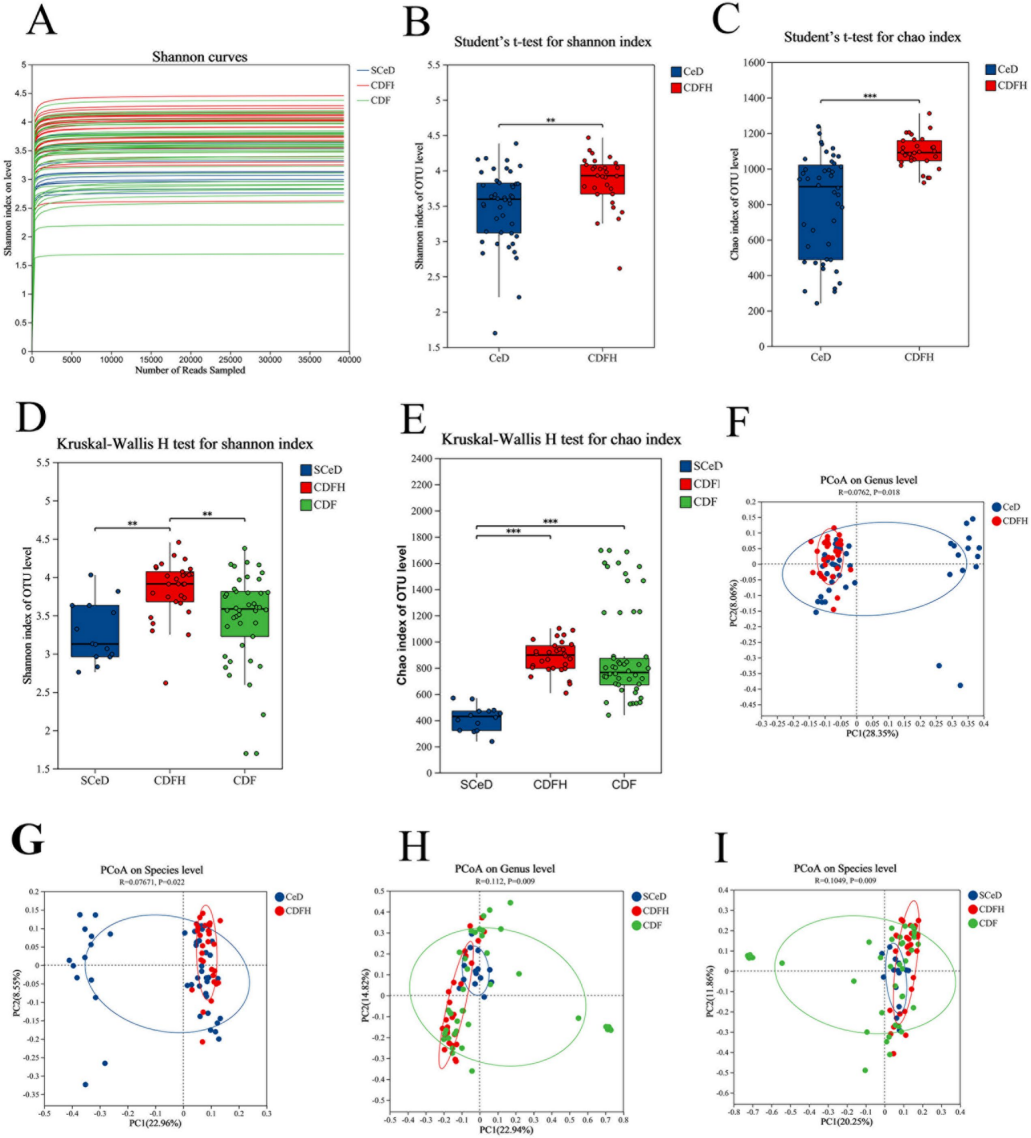
α-diversity and β-diversity. **(A)** Shannon diversity rarefaction curve. **(B)** Shannon index of the CeD group and CDFH group. **(C)** Chao index of the CeD group and CDFH group. **(D)** Shannon index of the SCeD group, CDF group, and CDFH group. **(E)** Chao index of the SCeD group, CDF group, and CDFH group. **(F)** β-diversity of the CeD group and CDFH group at the genus level. **(G)** β-diversity of the CeD group and CDFH group at the species level. **(H)** β-diversity of the SCeD group, CDF group, and CDFH group at the genus level. **(I)** β-diversity of the SCeD group, CDF group, and CDFH group at the species level.

For 16S rDNA sequencing, the generated sequences were clustered at the operational taxonomic unit (OTU) level with 97% similarity to reveal the composition of the gut microbiota. The Shannon diversity rarefaction curve tended to flatten (Figure 1A), indicating that the amount of sequencing data was appropriate and reasonable for representing the microbial community. In general, both the Shannon index and Chao index of the CeD group were lower than those of the CDFH group (Figures 1B,C). After dividing the disease group into different subtypes, the Sobs index of the SCeD group showed that the species richness of the SCeD group was lower than that of the other two groups (Supplementary Figures S1B,C). There was no significant difference in the Shannon index between the SCeD group and the CDF group, but both were significantly lower than that of the CDFH group (*p* < 0.05) (Figure 1D), which indicated that the species diversity of the disease groups was lower. The results of the Simpson index were consistent with those of the Shannon index. There was a significant difference in the Chao index between the SCeD group and the CDF group, with the SCeD group being lower than the CDF group (*p* < 0.05) (Figure 1E). Regarding the microbiota β-diversity, at the species and genus levels, there were certain differences in the principal coordinate analysis (PCoA) between the CeD group and the CDFH group (Figures 1F,G); there were significant differences in PCoA among the SCeD, CDF, and CDFH groups (Figures 1H,I).

**Figure 2:**
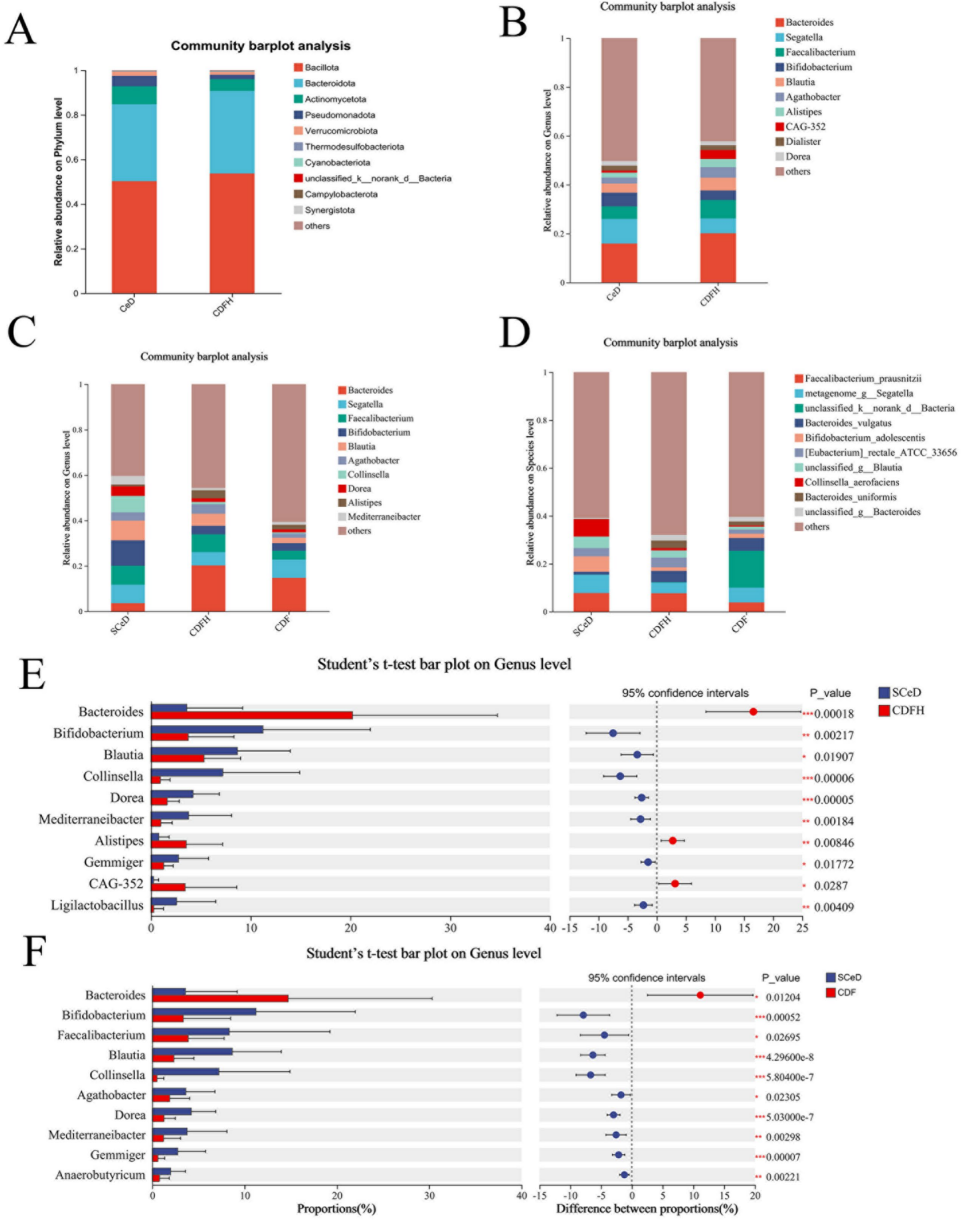
Microbial composition. **(A)** Phylum-level composition of the CeD group and CDFH group. **(B)** Genus-level composition of CeD and CDFH groups. **(C)** Phylum-level composition of SCeD, CDF, and CDFH groups. **(D)** Genus-level composition of SCeD, CDF, and CDFH groups. **(E)** Genus-level differential analysis between SCeD and CDFH groups. **(F)** Genus-level differential analysis between SCeD and CDF groups.

We compared the relative abundance of gut microbiota at the phylum and genus levels between the CeD group (CDF group and SCeD group) and the CDFH group. At the phylum level, compared with CDFH, the CeD group exhibited lower abundance of *Bacillota* and *Bacteroidota*, while showing higher abundance of *Actinomycetota* and *Pseudomonadota* (Figure 2A). At the genus level, compared with CDFH, the CeD group exhibited lower abundances of *Bacteroides*, *Faecalibacterium*, *Blautia*, *Agathobacter*, and *Alistipes*; while *Segatella* and *Bifidobacterium* showed higher abundances (Figure 2B).

To understand the relative abundance of microbiota in different types of CeD patients, we compared the abundance differences of gut microbiota at the phylum and genus levels among the SCeD, CDF, and CDFH groups. At the phylum level (Figure 2C), *Bacillota* exhibited the highest relative abundance across all three sample groups. Beyond *Bacillota*, *Actinomycetota* showed the highest abundance in the SCeD group. While *Bacteroidetota* dominated in CDF. At the genus level (Figure 2D), *Bacteroides* and *Faecalibacterium* exhibited high abundance in the CDFH group, suggesting strong adaptability and competitiveness in this environment. In the SCeD group, *Bifidobacterium*, *Blautia,* and *Faecalibacterium* were highly abundant. In the CDF group, *Bacteroides* and *Segatella* were highly abundant.

To identify microorganisms exhibiting significant differences between the two groups, we performed analysis using Student’s *t*-test. The results are shown in the figure. Compared to the CDFH group (Supplementary Figure S2), the CeD group exhibited higher abundances of Mediterranei-bacter, Streptococcus, and Lactobacillus species, while *Bacteroides*, *Agathobacter*, *Alistipes*, and others were lower than in the CDFH group. Compared to the CDFH group (Figure 2E), the SCeD group exhibited significantly lower levels of *Bacteroides*, *Alistipes*, and CAG-352; conversely, the SCeD group showed significantly higher levels of *Bifidobacterium*, *Blautia*, *Collinsella*, *Dorea*, *Mediterraneibacter*, *Gemmiger*, and *Lactobacillus* compared to the CDFH group (*p* < 0.05). Compared with the CDF group (Figure 2F), the SCeD group showed significantly lower levels of *Bacteroides* (*p* < 0.05) and significantly higher levels of *Bifidobacterium*, *Faecalibacterium*, *Blautia*, *Collinsella*, *Agathobacter*, *Dorea*, *Mediterraneibacter*, *Gemmiger*, and *Anaerobutyricum* (*p* < 0.05).

### 4.2 Metabolomics analysis results

#### 4.2.1 Metabolites significantly altered during the development of CeD and its subtypes

**Figure 3:**
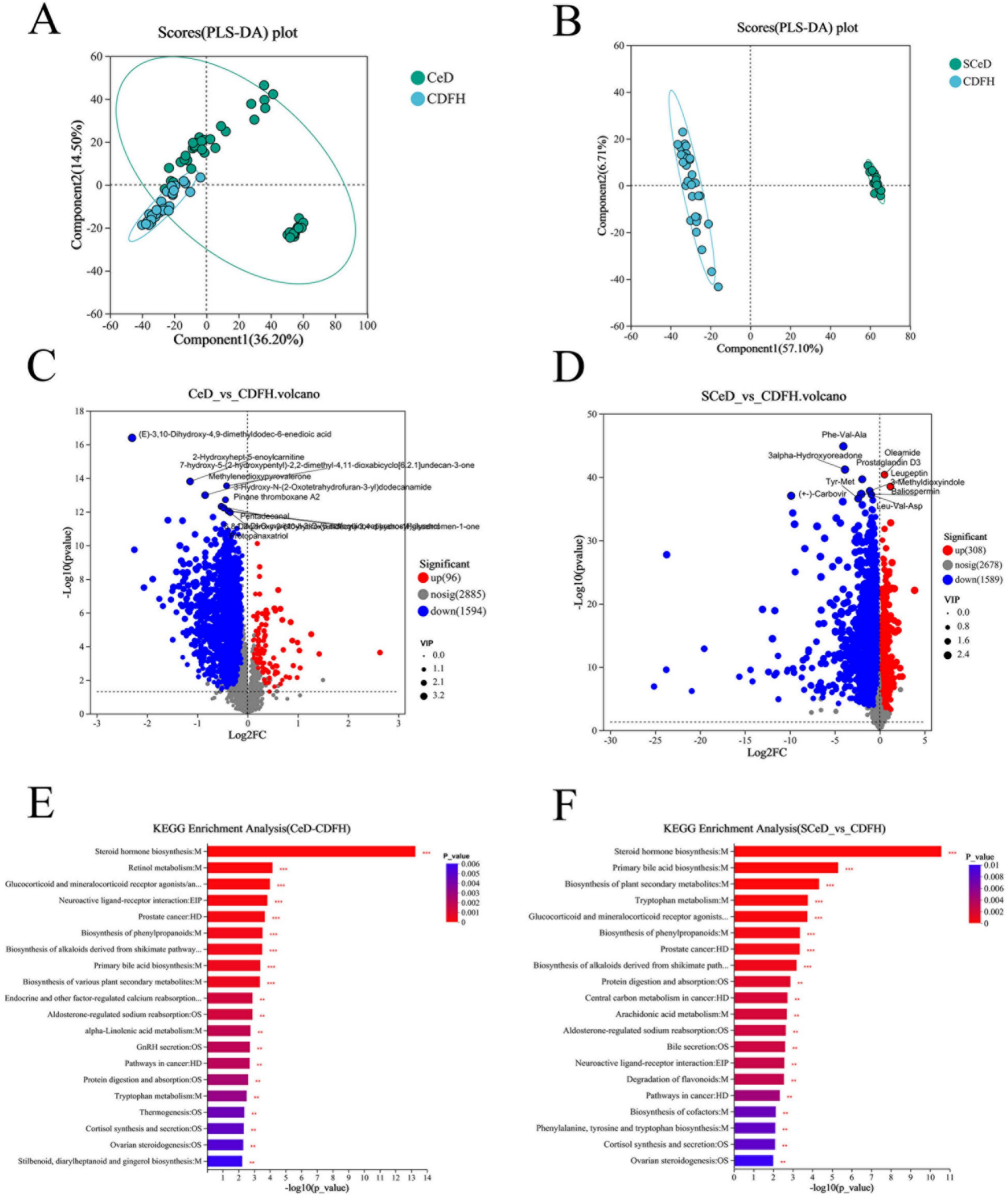
Metabolite changes in CeD patients. **(A)** PLS-DA scores of the CeD group vs. the CDFH group. **(B)** PLS-DA scores of the SCeD group vs. the CDFH group. **(C)** Volcano plot of differential metabolites between the CeD group and the CDFH group. **(D)** Volcano plot of differential metabolites between the SCeD group and the CDFH group. **(E)** KEGG enrichment analysis of differential metabolites between the CeD group and the CDFH group. **(F)** KEGG enrichment analysis of differential metabolites between the SCeD group and the CDFH group.

Metabolite characteristics among the groups are shown in Supplementary Figure S3. There were 1,543 shared metabolites among the CDFH, CDF, and SCeD groups; the number of unique metabolites in each group was 435, 92, and 4,170, respectively. There were both shared and differential metabolites among the groups, with specific details provided in Supplementary Tables. The partial least squares discriminant analysis (PLS-DA) model of the CeD and CDFH groups indicated differences in metabolites between the two groups (Figure 3A). The volcano plot showed that under the conditions of *p* < 0.05 and VIP > 1, there were 1,690 differentially expressed metabolites (96 upregulated and 1,594 downregulated) (Figure 3C). Through KEGG analysis, the differential metabolites were mainly enriched in pathways such as Steroid hormone biosynthesis, Retinol metabolism, and Neuroactive ligand-receptor interaction (Figure 3E), suggesting that these key metabolic functions are abnormal in CeD patients. The PLS-DA model of the SCeD and CDFH groups revealed significant differences in metabolites between the two groups (Figure 3B). The volcano plot showed that under the conditions of *p* < 0.05 and VIP > 1, there were 1,897 differentially expressed metabolites (308 upregulated and 1,589 downregulated) (Figure 3D). KEGG analysis indicated that these differential metabolites were mainly enriched in pathways including Steroid hormone biosynthesis, Primary bile acid biosynthesis, and Tryptophan metabolism (Figure 3F), suggesting that compared with the CDFH group, these key metabolic functions are abnormal in SCeD patients. These analyses indicate that Steroid hormone biosynthesis may play an important role in the development of CeD.

The x-axis represents the fold change value of metabolite expression difference between the two groups, i.e., log2FC; the y-axis represents the statistical test value of the difference in metabolite expression level change, i.e., −log10(p_value). The higher the value, the more significant the expression difference.

#### 4.2.2 Relationship between different types of CeD and metabolites

**Figure 4:**
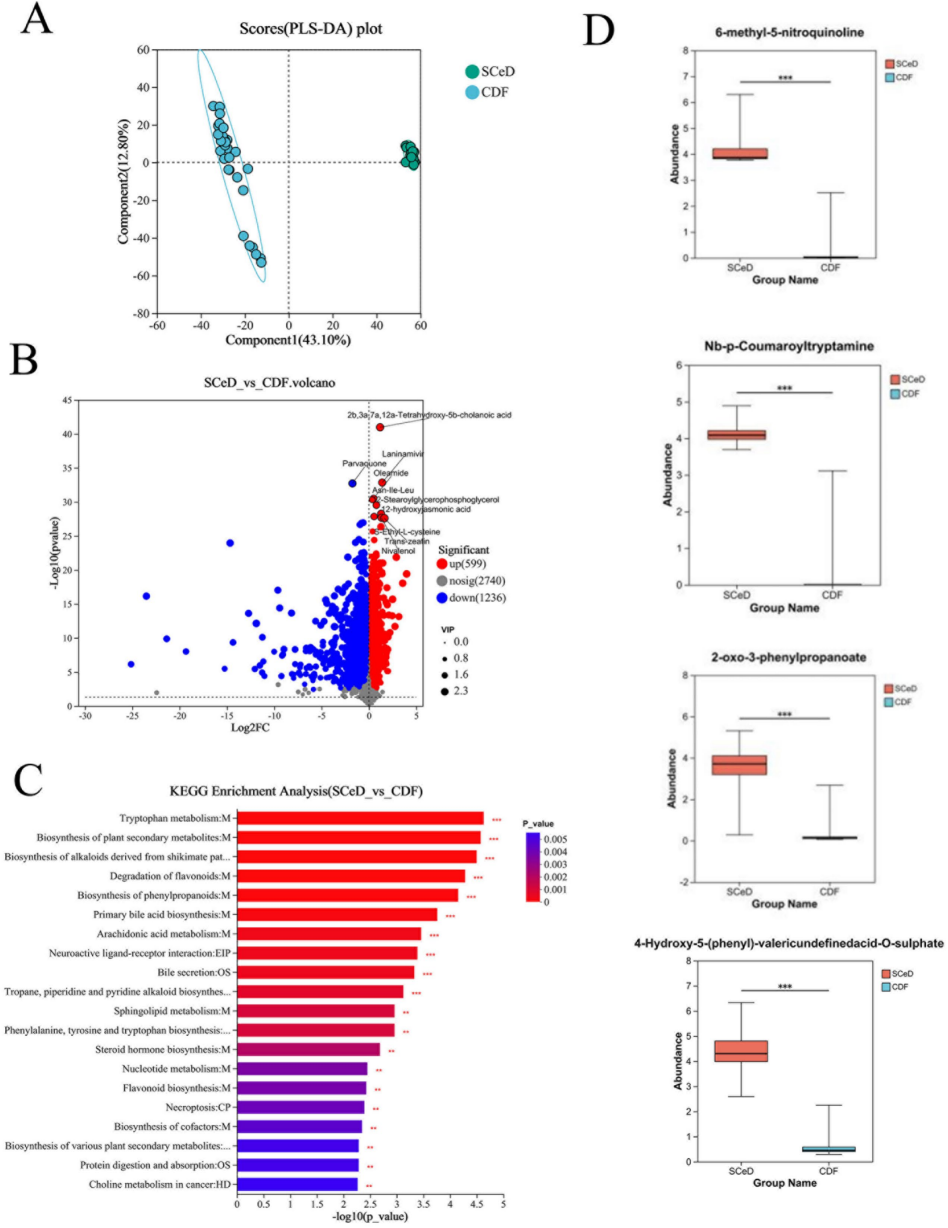
Differences in metabolite types between CeD groups. **(A)** PLS-DA scores for SCeD and CDF groups. **(B)** Volcano plot of differential metabolites between SCeD and CDF groups. **(C)** KEGG analysis of differential metabolites between SCeD and CDF groups. **(D)** Top four differential metabolites with the highest fold change in SCeD relative to CDF groups.

The PLS-DA model results for the SCeD and CDF groups revealed significant differences in metabolites between the two groups (Figure 4A). Under the conditions of *p* < 0.05 and VIP > 1, 1,835 differentially expressed metabolites were identified (599 upregulated and 1,236 downregulated) (Figure 4B). KEGG analysis revealed that differentially expressed metabolites were primarily enriched in pathways including Tryptophan metabolism, Biosynthesis of plant secondary metabolites, Biosynthesis of alkaloids derived from shikimate pathway, and Degradation of flavonoids (Figure 4C). Compared to the CDF group, 6-methyl-5-nitroquinoline (FC = 15.8745), Nb-p-coumaroyltryptamine (FC = 11.9082), 2-oxo-3-phenylpropanoate (FC = 9.0352), and 4-hydroxy-5-(phenyl)-valeric acid-O-sulfate (FC = 7.4546) were significantly upregulated in the SCeD group with large fold changes (Figure 4D).

### 4.3 Construction of diagnostic models for CeD and SCeD based on differential microorganisms and metabolites

**Figure 5:**
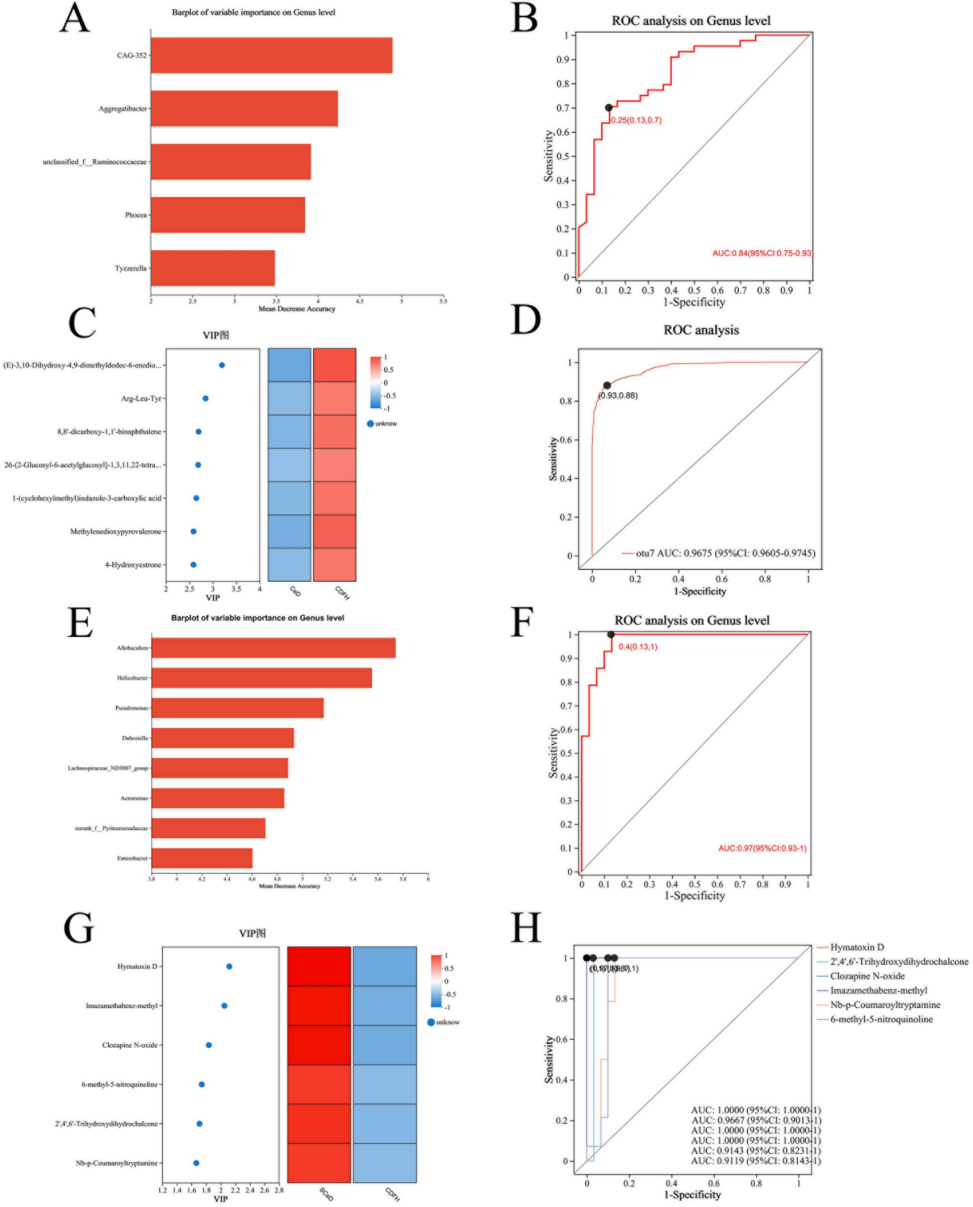
Diagnostic Models Based on Differential Microorganisms and Metabolites. **(A)** Top 5 differential microorganisms (ranked by importance) between the CeD group and CDFH group. **(B)** ROC curve of the 5 differential microorganisms for CeD. **(C)** Top 7 differential metabolites between the CeD group and CDFH group. **(D)** ROC curve of the 7 differential metabolites for CeD. **(E)** Top 8 differential microorganisms (ranked by importance) between the SCeD group and CDFH group. **(F)** ROC curve of the 8 differential microorganisms for SCeD. **(G)** Top 6 differential metabolites between the SCeD group and CDFH group. **(H)** ROC curve of the 6 differential metabolites for SCeD.

We developed a diagnostic model for CeD (SCeD and CDF) based on differential microorganisms and metabolites. To investigate the diagnostic value of microorganisms in CeD, we employed the random forest model to identify candidate microbial taxa for achieving the optimal AUC value. Through feature selection with 10-fold cross-validation, we ultimately selected five candidate microbial taxa to construct the diagnostic model for CeD (AUC = 0.84, Figures 5A,B), indicating that these five taxa hold potential as diagnostic biomarkers for CeD. To investigate the diagnostic value of metabolites for CeD, we selected the top 7 differential metabolites (ranked by species importance) based on the random forest model to construct a diagnostic model for CeD. Each of these 7 differential metabolites individually exhibited high diagnostic value for CeD (AUC ≥ 0.85, see Supplementary Figure S4 for details), and the diagnostic model built by combining these 7 differential metabolites achieved an AUC of 0.96 (Figures 5C,D). In addition, we constructed a combined diagnostic model based on both differential microorganisms and differential metabolites, which significantly improved the diagnostic value for CeD (AUC = 0.98, Supplementary Figure S5). These results indicate that the CeD diagnostic models constructed based on either differential microorganisms or metabolites both have good diagnostic value. However, the current sample size is limited, and their diagnostic efficacy has not been validated in larger-scale, more diverse clinical cohorts. Therefore, these models are not yet ready for direct application in clinical testing.

To explore the diagnostic ability of differential microorganisms and differential metabolites for SCeD, we performed random forest regression analysis based on differential microorganisms and used the top 8 microorganisms (ranked by importance) to establish a diagnostic model. The combined diagnostic model constructed based on these 8 microorganisms achieved an AUC of 0.97 (Figures 5E,F), indicating excellent diagnostic efficacy of this model—i.e., these 8 microorganisms have the potential to serve as potential biomarkers for SCeD. Furthermore, based on the VIP values of differential metabolites between the two groups, we selected the top 6 metabolites (ranked by VIP values) to establish a diagnostic model (AUC ≥ 0.9, Figures 5G,H). The AUC values of Hymatoxin D, 2′,4′,6′-Trihydroxydihydrochalcone, Clozapine N-oxide, Imazamethabenz-methyl, Nb-p-Coumaroyltryptamine, and 6-methyl-5-nitroquinoline were 1, 0.9667, 1, 1, 0.9143, and 0.9119, respectively. The results indicated that these metabolites may have the ability to distinguish between SCeD and CDFH. In conclusion, these differential microorganisms and metabolites exhibit good discriminatory potential for the SCeD group.

### 4.4 Correlation analysis of differential microorganisms and differential metabolites

**Figure 6:**
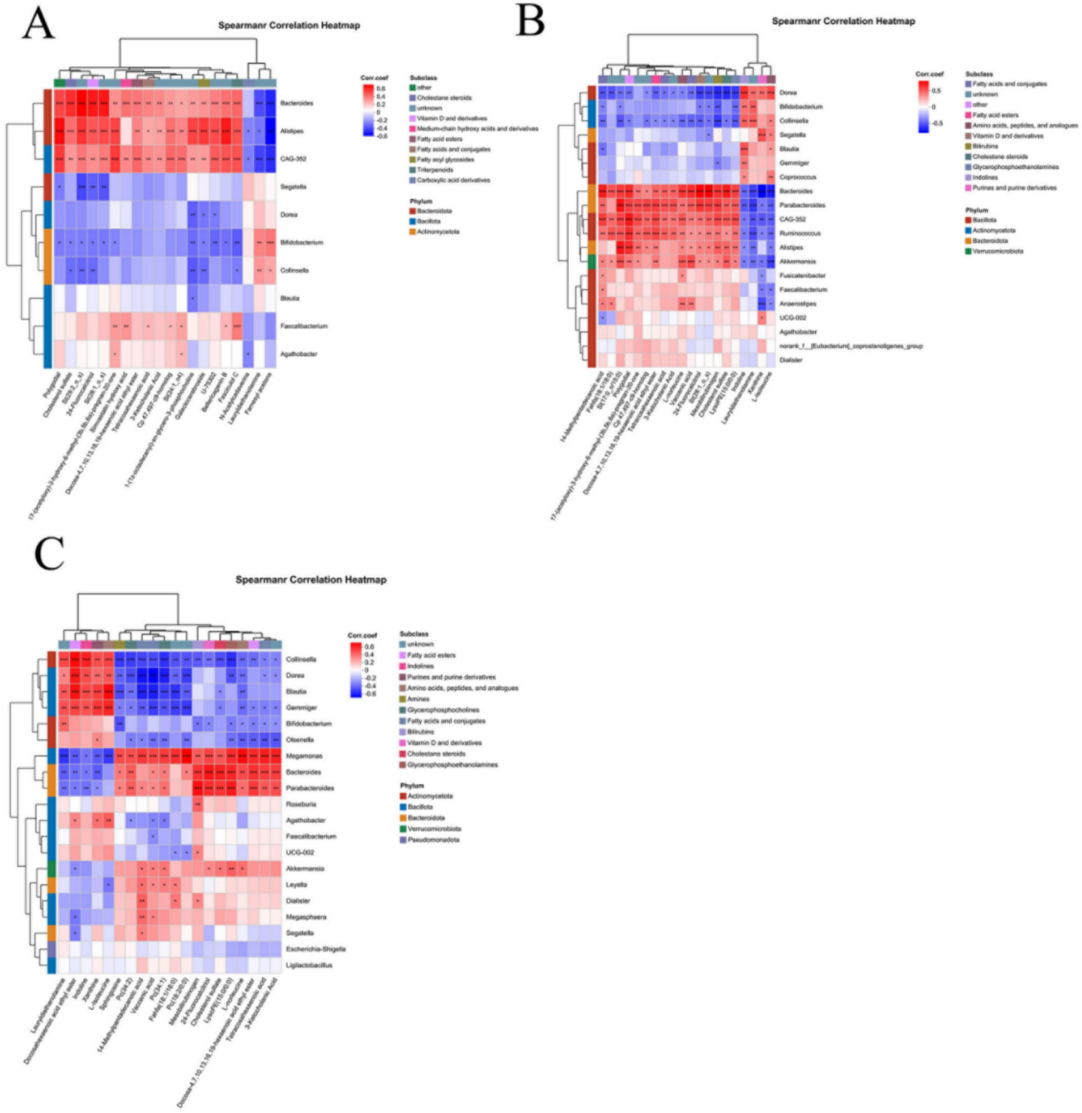
Correlation analysis of differential microbes and differential metabolites. **(A)** Correlation between CeD and CDFH groups. **(B)** Correlation between SCeD and CDFH groups. **(C)** Correlation between SCeD and CDF groups.

Given the significant differences in microbial and metabolite levels across subgroups, and considering that combining these factors enhances diagnostic performance, analyzing correlations between microorganisms and metabolites is of great significance. Spearman correlation analysis revealed correlations between the top 20 differentially abundant microorganisms and metabolites, as shown in Figure 6. Correlation analysis between CeD and CDFH groups revealed that *Bacteroides* and *Alistipes* were positively correlated with Docosa-4,7,10,13,16,19-hexaenoic acid ethyl ester, Tetracosahexaenoic acid, and 3-Ketocholanic Acid; and negatively correlated with N-Acetylcadaverine, Lauryldiethanolamine, and Farnesyl acetone (Figure 6A). Spearman correlation analysis between the SCeD group and CDFH group revealed consistent relationships between *Bacteroides* and CAG-352 with metabolites: both showed significant positive correlations with multiple metabolites including Vaccenic acid, 3-Ketocholanic Acid, and Docosa-4,7,10,13,16,19-hexaenoic acid ethyl ester; while negatively correlated with Lauryldiethanolamine, Xanthine, and L-Isoleucine*. Bifidobacterium*, *Collinsella*, and *Dorea* showed positive correlations with Indoline and Lauryldiethanolamine, and negative correlations with Mesobilirubinogen, Vaccenic acid, and St (28:1_o_s) (Figure 6B). In both SCeD and CDF groups, *Bacteroides* showed positive correlations with Mesobilirubinogen, Cholesterol sulfate, and Tetracosahexaenoic acid; and negative correlations with Docosahexaenoic acid ethyl ester, L-Isoleucine, and Lauryldiethanolamine. Microorganisms such as *Collinsella*, *Dorea*, and *Gemmiger* were positively correlated with Docosahexaenoic acid ethyl ester, L-Isoleucine, and Lauryldiethanolamine; negatively correlated with 14-Methylpentadecanoic acid, Docosa-4,7,10,13,16,19-hexaenoic acid ethyl ester, and Tetracosahexaenoic acid (Figure 6C).

## 5 Discussion

In this study, we analyzed 16 s rDNA sequencing and LC–MS data from 74 fecal samples and identified differences in various microorganisms and metabolites across comparative groups. Integrated multi-omics analysis revealed specific microbes and metabolites associated with the occurrence and symptoms of CeD. The multi-omics integration approach helped uncover biologically relevant pathways in CeD, while machine learning enabled the identification of multiple biomarkers capable of effectively distinguishing the SCeD group from the CDFH group. Furthermore, our results demonstrated differences in microorganisms and metabolites between the symptomatic CeD group (CDF) and the asymptomatic CeD group (SCeD), indicating the important role of microbes and metabolites in driving CeD symptoms.

At the phylum level, *Firmicutes* accounted for the highest proportion across all groups, which is consistent with previous CeD studies reporting *Firmicutes* as the dominant phylum in fecal microbiota (Constante et al., 2022). Apart from *Firmicutes*, the SCeD group showed a higher proportion of *Actinobacteria*. *Actinobacteria* are Gram-positive bacteria widely distributed in nature and exhibit a dual functional nature. *Bifidobacteria*, representative probiotics within this phylum, can protect the intestine by modulating immune responses and enhancing the intestinal barrier (Al-Sadi et al., 2021; Zhou et al., 2024). However, the genus Actinomyces constitutes 55–68% of Actinobacteria and, along with its related metabolites, plays a pathogenic role in genitourinary tract infections and actinomycosis (Gajdács and Urbán, 2020; Könönen and Wade, 2015). Additionally, one study reported elevated levels of *Actinobacteria* in cancer groups of unknown primary origin (Dorobisz et al., 2024). Another study indicated that CeD patients had relatively higher proportions of *Firmicutes* and *Actinobacteria* compared to treated subjects [68]. These findings suggest that *Actinobacteria* may be associated with the development and progression of CeD.

At the genus level, compared with the CDFH group, the CeD group exhibited lower abundances of *Bacteroides*, *Faecalibacterium*, *Blautia, Agathobacter*, and *Alistipes*, and higher abundances of *Segatella* and *Bifidobacterium*. The change in *Bacteroides* abundance was consistent with the findings of a previous study (Di Biase et al., 2021). Studies have shown that *Bacteroides* can enhance intestinal barrier function, regulate immunity, and modulate inflammation by producing short-chain fatty acids (SCFAs) (Brown et al., 2019); thus, immune dysregulation and intestinal barrier dysfunction in CeD patients may be closely associated with *Bacteroides*. However, other studies have indicated that *Bacteroides* dominates the duodenal microbiota in active CeD (Lupu et al., 2023). Such discrepancies among different studies may be closely related to disease status and sample type. The changes in *Faecalibacterium* and *Blautia* were also consistent with previous research, showing a significant decrease in their abundances in CeD (Shi et al., 2022). *Agathobacter* abundance was higher in groups with high genetic risk (HLA DQ2/DQ8 positive) and positive serum antibodies, while *Alistipes* abundance was higher in non-high-risk groups (Aguayo-Patrón et al., 2023). Additionally, studies have reported that *Agathobacter*—a genus that produces SCFAs—is reduced in CeD (Jing et al., 2023), which is consistent with the results of our study. The mechanism underlying the role of *Segatella* in CeD remains unclear. However, studies have shown that *Segatella* abundance is increased in rheumatoid arthritis (RA), and its potential role in the pathogenesis of RA may involve stimulating helper T cell (Th) 17 populations and inducing the production of Th17 cell-related cytokines (IL-6 and IL-23) (Korzeniowska et al., 2024). IL-6, IL-23, and Th17 cells have also been reported in CeD-related studies (Fryk et al., 2024). Therefore, the presence of CeD-related inflammatory factors may be associated with *Segatella.*

Compared with the CDFH group, the SCeD group exhibited significantly lower abundances of *Bacteroides*, *Alistipes*, and CAG-352, and significantly higher abundances of genera including *Bifidobacterium, Blautia, Collinsella, Dorea, Mediterraneibacter, Gemmiger,* and *Ligilactobacillus*. Since no detailed reports on the microbiota of SCeD patients have been published previously, we can only provide a possible explanation for the development of SCeD based on the inherent functions of the bacteria themselves. Some bacteria with anti-inflammatory effects (*Blautia, Gemmiger*) were more abundant in the SCeD group; it is hypothesized that the microbiota may compensate for the host’s physiological imbalance by increasing probiotics, thereby alleviating the occurrence of symptoms (Chandrasekaran et al., 2024). Studies have shown that the abundance of *Bacteroides* is lower in patients with inflammatory bowel disease (IBD) (Zhou and Zhi, 2016), and supplementation with *Bacteroides thetaiotaomicron* and its inactivated form can alleviate colitis by inhibiting the activation of macrophages (Yinhe et al., 2024). Research indicates that insufficient dietary fiber intake may lead to the overgrowth of *Collinsella*, alter the overall fermentation pattern of the gut microbiota, and exert potential adverse effects on the host’s metabolic and inflammatory health (Gomez-Arango et al., 2018). However, other studies have shown that ursodeoxycholic acid produced by *Collinsella* can inhibit cytokine storm syndrome and prevent COVID-19 infection (Hirayama et al., 2021). It is evident that *Collinsella* plays different roles in different diseases; it may exert a beneficial effect in SCeD, and further research is needed to confirm this. Studies have reported that *Mediterraneibacter* is enriched in fecal samples from mice with polycystic ovary syndrome (PCOS), as well as in samples from patients with irritable bowel syndrome (IBS) and colorectal polyps (Huang F. et al., 2024; Intarajak et al., 2024; Jagare et al., 2023). The abundance of *Escherichia-Shigella* is increased in various diseases, such as chronic pancreatitis, pancreatic ductal adenocarcinoma (PDAC), autism spectrum disorder (ASD), and inflammatory bowel disease (IBD) (Chen et al., 2024; Hong et al., 2024; Valenzuela-Zamora et al., 2022). In conclusion, the development and specificity of SCeD may be the result of interactions among multiple microorganisms. However, the complex and diverse interactions require support from more basic experiments.

Compared with the CDF group, the SCeD group exhibited a significantly lower abundance of *Bacteroides*, and significantly higher abundances of *Bifidobacterium*, *Faecalibacterium*, *Blautia*, *Collinsella, Agathobacter, Dorea*, *Mediterraneibacter*, *Gemmiger,* and *Anaerobutyricum.* This corresponds with findings from other studies indicating that Bacteroides is associated with inflammation, while *Bifidobacterium* and *Faecalibacterium* possess anti-inflammatory properties (Charlet et al., 2020; Huang Y. et al., 2024; Quévrain et al., 2016). The abundances of *Faecalibacterium* and *Agathobacter* were significantly higher in the SCeD group than in the CDF group. Studies have shown that butyrate— a fermentation product of *Faecalibacterium prausnitzii*, a key member of the *Faecalibacterium* genus—exerts anti-inflammatory effects via the NOD2-mediated signaling pathway. It promotes the expression of anti-inflammatory cytokines (e.g., IL-10 and IFN-γ), inhibits the expression of pro-inflammatory cytokines (e.g., IL-12), and exerts a protective effect on the intestinal mucosa (Touch et al., 2022). Furthermore, our analysis at the species level revealed that the abundance of *Faecalibacterium prausnitzii* was significantly higher in the SCeD group than in the CDF group. Therefore, the high abundance of *Faecalibacterium prausnitzii* may inhibit pro-inflammatory cytokine expression, promote anti-inflammatory cytokine expression, and enhance immune responses—thereby preventing the development of relevant clinical symptoms in SCeD patients. Studies have reported that *Agathobacter* and its metabolic product butyrate can alleviate neuroinflammation induced by Alzheimer’s disease (AD) by regulating the NF-κB signaling pathway (Lv et al., 2024). Additionally, earlier studies have identified NF-κB as a potential molecular target for regulating inflammatory responses in celiac disease (CeD) (Maiuri et al., 2003). In conclusion, the increased abundances of *Faecalibacterium* and *Agathobacter* may be important contributing factors to the absence of clinical symptoms in SCeD patients. The results of this sequencing data will provide a certain theoretical basis for subsequent experiments.

In metabolomic studies, it has been found that changes in metabolites are associated with a variety of diseases (Yuan et al., 2024), such as inflammatory bowel disease (IBD), type 1 diabetes mellitus (T1DM), and systemic lupus erythematosus (SLE) (Huang et al., 2022; Li et al., 2024; Vich Vila et al., 2023; Zeng et al., 2022); however, there are relatively few metabolomic studies on SCeD. In the present study, through LC–MS analysis, we found that the SCeD group exhibits unique metabolic profiles compared with the CDFH group and CeD group. Compared with the CDFH group, 1,897 metabolites were differentially expressed in the SCeD group (*p* < 0.05, VIP > 1). Through KEGG analysis, these differential metabolites were mainly enriched in pathways such as Steroid hormone biosynthesis, Primary bile acid biosynthesis, and Tryptophan metabolism. Under the conditions of *p* < 0.05 and VIP > 2, there were 44 differentially expressed metabolites (4 upregulated and 40 downregulated). The four upregulated metabolites—Tetrahydrodeoxycortisol, Hymatoxin D, Imazamethabenz-methyl, and Ile-Asp-may play a role in the development and progression of SCeD.

Compared with the CDF group, a total of 1,835 metabolites were differentially expressed between the SCeD group and the CDF group (*p* < 0.05, VIP > 1). Through KEGG analysis, these differential metabolites were mainly enriched in pathways such as Tryptophan metabolism, Biosynthesis of plant secondary metabolites, Biosynthesis of alkaloids derived from the shikimate pathway, and Degradation of flavonoids. Existing studies have shown that tryptophan metabolism is altered in CeD patients, and the score of the Gastrointestinal Symptom Rating Scale (GSRS) was also significantly reduced after tryptophan intervention (Asgari et al., 2025). In our study, the abundance of (+/−)-Tryptophan in the SCeD group was higher than that in the CDF group. Additionally, studies have indicated that tryptophan metabolites regulate intestinal barrier function via the aryl hydrocarbon receptor (AhR) (Lamas et al., 2020). Furthermore, research has suggested that tryptophan has the potential to treat CeD by regulating immune responses (Asgari et al., 2025). This also provides certain evidence supporting the role of tryptophan metabolism in the clinical symptoms of CeD. Combined with the microbial findings, flavonoids can be converted into short-chain fatty acids (SCFAs) by a variety of bacteria, and the converted SCFAs improve the intestinal barrier through anti-inflammatory effects (Al-Khayri et al., 2022; Liu et al., 2021). Compared with the CDF group, the abundances of 6-methyl-5-nitroquinoline, Nb-p-Coumaroyltryptamine, 2-oxo-3-phenylpropanoate, and 4-Hydroxy-5-(phenyl)-valeric acid-O-sulfate were higher in the SCeD group.6-methyl-5-nitroquinoline contains a quinoline structure and may be associated with the aryl hydrocarbon receptor (AhR) pathway. AhR is a transcription factor that can be activated by a variety of ligands; activated AhR can promote the differentiation of regulatory T cells (Treg cells), and Treg cells possess immunosuppressive functions that can alleviate inflammatory responses (Zeng et al., 2022). 4-Hydroxy-5-(phenyl)-valeric acid-O-sulfate is a compound related to flavan-3-ol metabolism. Some studies have shown that flavan-3-ols and their metabolites have anti-inflammatory activity (Yang Q. et al., 2022). Multiple studies have demonstrated that changes in microorganisms and metabolites can protect the intestinal barrier by regulating the inflammatory state (Gilsenan et al., 2024; Li S. et al., 2023).

Based on the random forest model, we found that differential microorganisms and differential metabolites not only exhibited excellent diagnostic performance for CeD (the microbial-based model achieved an AUC of 0.84, the metabolite-based model an AUC of 0.96, and the combined model of differential microorganisms and metabolites an AUC of 0.98) but also showed good diagnostic performance for SCeD (the microbial-based model achieved an AUC of 0.97, and the metabolite-based model an AUC ≥ 0.9). However, given the small sample size in our study, their diagnostic efficacy (e.g., sensitivity and specificity) still needs to be validated in larger-scale and more diverse clinical cohorts, and the models are not yet ready for direct application in clinical testing. Nevertheless, this also suggests that microorganisms and metabolites have the potential to serve as diagnostic biomarkers for SCeD. Multiple studies have indicated that changes in fecal metabolites are associated with gut microbiota during disease progression, such as in ulcerative colitis (UC), metabolic-associated fatty liver disease (MAFLD), and primary Sjögren’s syndrome (pSS) (Yang et al., 2022a,b; Yang et al., 2021). In this study, via Spearman correlation analysis, we identified a certain correlation between differential microorganisms and differential metabolites.

This study also has certain limitations. First, the sample size included in the study is small, with only 14 cases in the SCeD group, which is insufficient to fully reflect the changes in gut microbiota and metabolites in SCeD patients. Future studies should expand the sample size of SCeD patients to explain the unique gut microbiota and metabolic characteristics of these patients.

Second, CeD is a heterogeneous disease, encompassing classic CeD, atypical CeD, and SCeD. In this study, we only compared the differences in microbiota and metabolites between the CDF group and the SCeD group, while the gut microbiota and metabolomic characteristics of other CeD subtypes were not reflected. Additionally, gut microbiota and metabolomic results are influenced by factors such as environment, diet, and geography. All patients included in this study were from Xinjiang, China, and Xinjiang has its unique geographical and dietary environments—this may result in the lack of generalizability of the study’s findings. In the future, larger-sample longitudinal studies can be conducted, with the incorporation of dietary data, to validate the current research results and control for confounding variables.

## 6 Conclusion

We detected the microbiota and metabolites in fecal samples from SCeD patients using 16S rDNA sequencing and liquid chromatography-mass spectrometry (LC–MS). The results showed that there were differences in microbiota and metabolites between the CeD group (including SCeD and CDF) and the CDFH group, between the SCeD group and the CDFH group, and between the SCeD group and the CDF group. In addition, correlation analysis revealed a correlation between changes in differential microorganisms and differential metabolites across different groups. In this study, we found that both the composition and abundance of gut microbiota and metabolites in CeD patients had changed. Patients with different CeD subtypes (SCeD and CDF) exhibited unique fecal microbiota and metabolite characteristics. Therefore, changes in microorganisms and metabolites not only participate in the development of CeD but also are associated with different subtypes of CeD. In conclusion, the pathogenesis of different CeD subtypes may involve a “host-microbe-metabolite” trinity interaction network. Based on machine learning, a random forest model can be established using differential microorganisms and metabolites between the SCeD group and the CDFH group, serving as a diagnostic tool for SCeD. Additionally, modulating the differential microorganisms and metabolites across groups can act as a new entry point for studying the mechanism of CeD and an adjunctive approach for its treatment.

## Data Availability

The data presented in the study are deposited in the NCBI repository (https://www.ncbi.nlm.nih.gov/), The accession number for the typical celiac disease group and healthy control group is PRJNA890948, and the accession number for asymptomatic celiac disease is PRJNA1354235.

## Footnotes

^1^ https://cloud.majorbio.com
