# Supplementary material for: Integrated analysis of fecal microbiota and metabolomics reveals unique characteristics of asymptomatic and classic celiac disease
Source: Front Microbiol. 2025 Nov 13;16:1636007. doi: 10.3389/fmicb.2025.1636007 (PMC12659859; doi:10.3389/fmicb.2025.1636007)
Supplement: Supplementary file 5 [file Supplementary_file_1.docx]

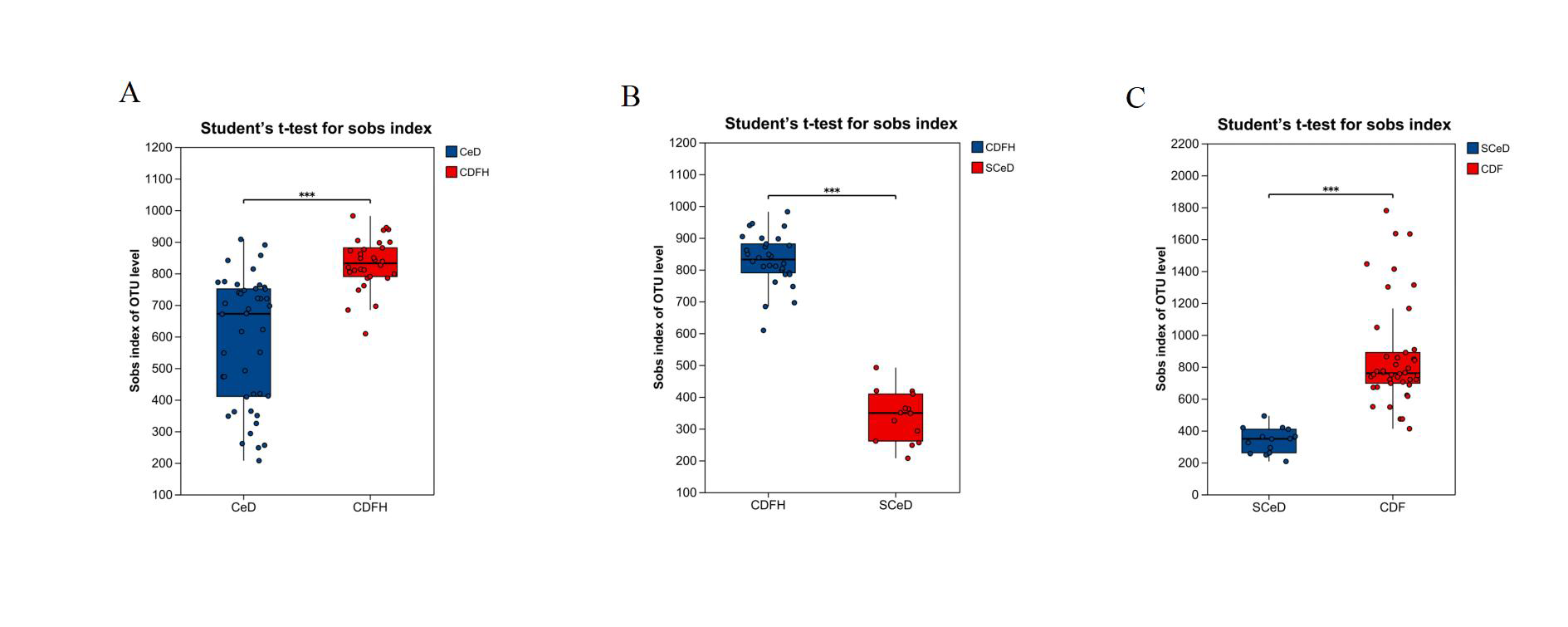


**Supplementary Figure 1 Alpha diversity**(A) Sobs index between CeD and CDFH groups.(B) Sobs index between SCeD and CDFH groups.(C) Sobs index between SCeD and CDF groups.


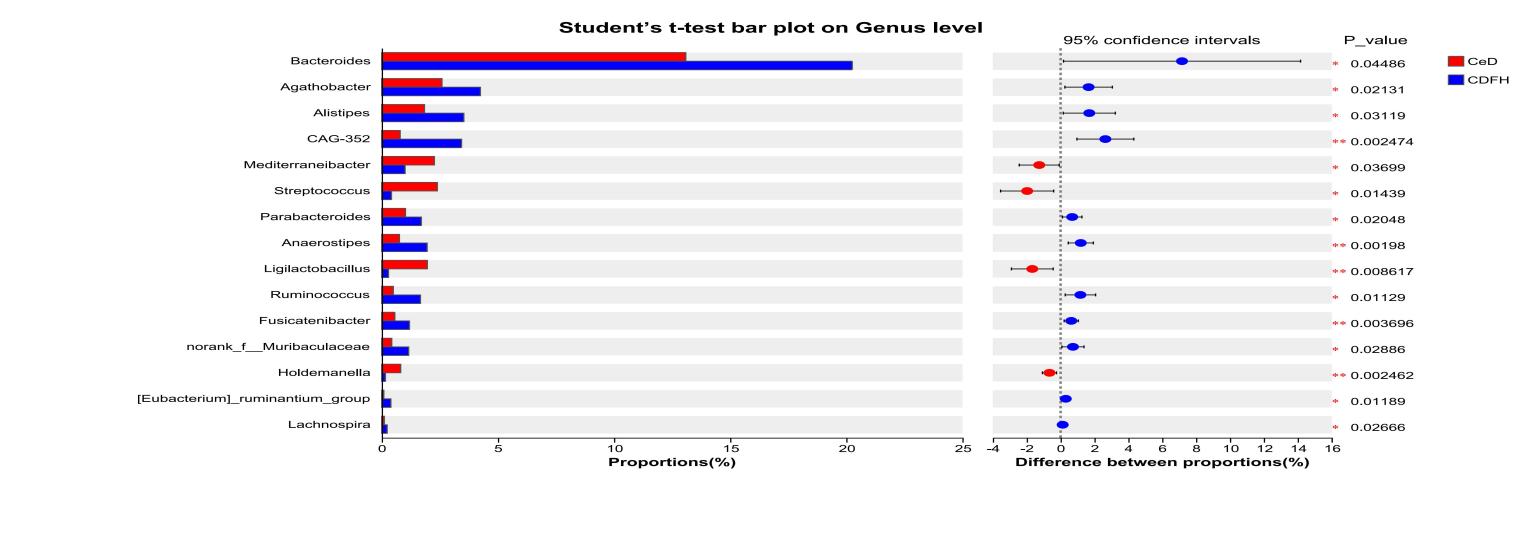


Supplementary Figure 2. Microbial differences between the CeD group and the CDFH group at the genus level


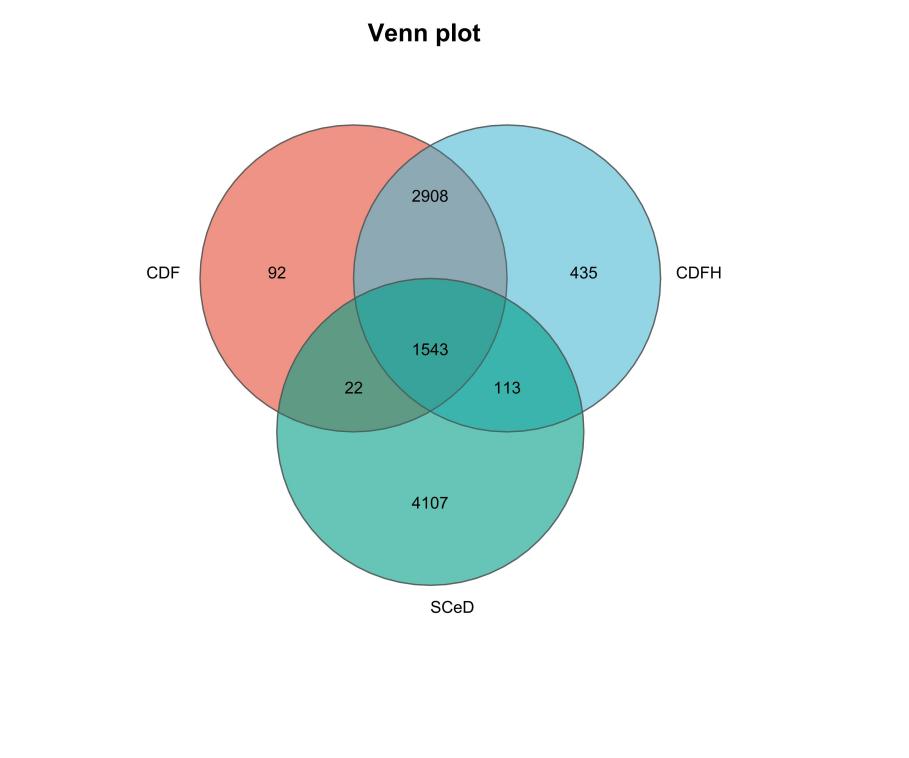


Supplementary Figure 3: Total and unique metabolite types in the SCeD, CDF, and CDFH groups


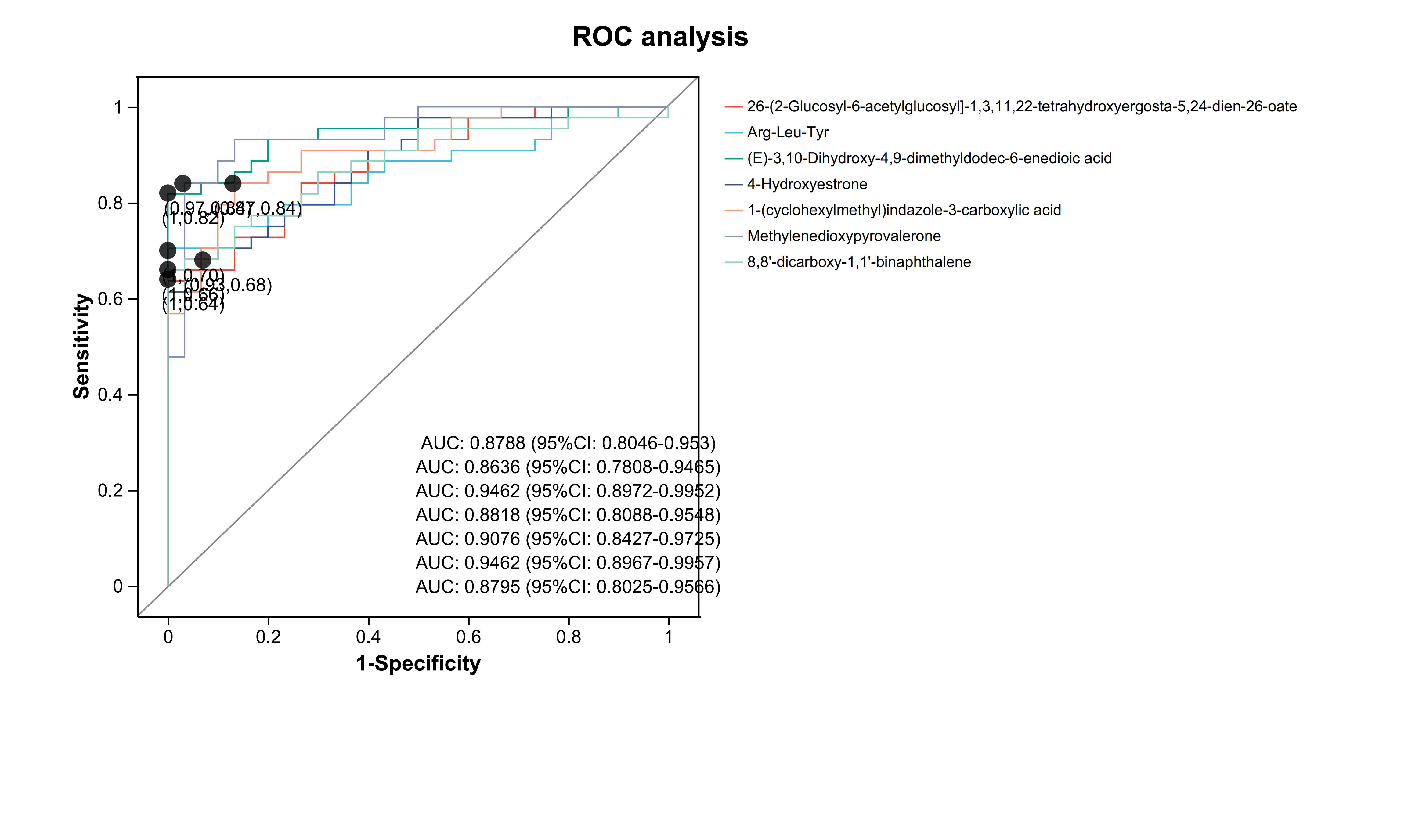


Supplementary Figure 4 Diagnostic model based on differential metabolites.**The diagnostic value of individual metabolites for CeD is shown in the figure.**


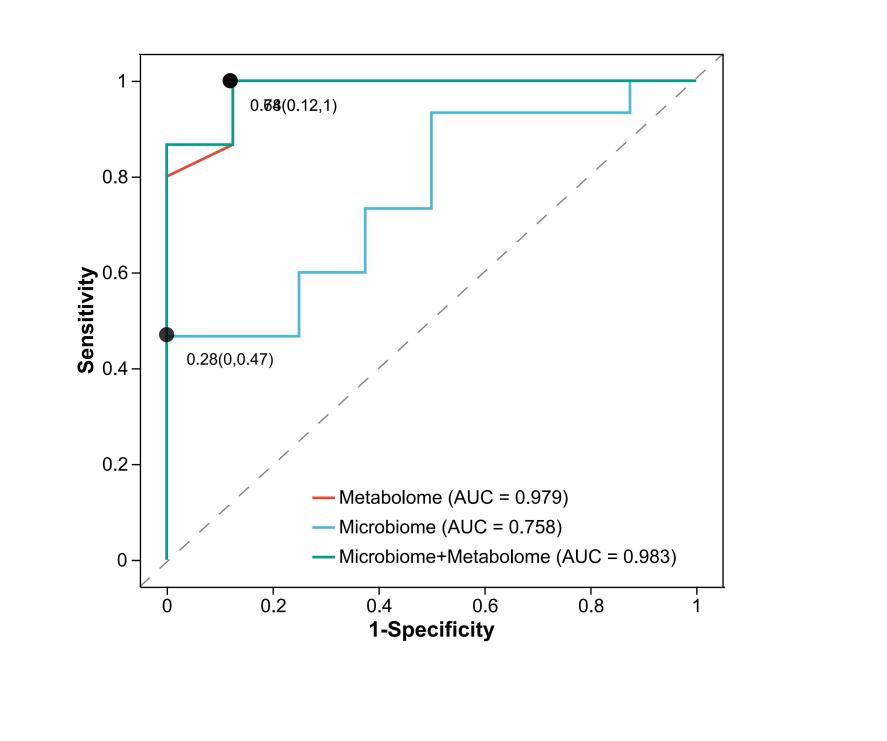


Supplementary Figure 5 The combined use of differential microorganisms and differential metabolites in the diagnosis of CeD
